# Disparities in telemedicine during COVID‐19

**DOI:** 10.1002/cam4.4518

**Published:** 2022-01-05

**Authors:** Alexander S. Qian, Melody K. Schiaffino, Vinit Nalawade, Lara Aziz, Fernanda V. Pacheco, Bao Nguyen, Peter Vu, Sandip P. Patel, Maria Elena Martinez, James D. Murphy

**Affiliations:** ^1^ Department of Radiation Medicine and Applied Sciences University of California San Diego La Jolla California USA; ^2^ School of Public Health Division of Health Management and Policy San Diego State University San Diego California USA; ^3^ Center for Health Equity, Education and Research (CHEER) University of California San Diego La Jolla California USA; ^4^ Department of Medicine Division of Hematology‐Oncology University of California San Diego La Jolla California USA; ^5^ University of California San Diego Herbert Wertheim School of Public Health and Human Longevity Science La Jolla California USA

**Keywords:** community outreach, ethical considerations, medical oncology, QOL

## Abstract

**Background:**

Oncology rapidly shifted to telemedicine in response to the COVID‐19 pandemic. Telemedicine can increase access to healthcare, but recent research has shown disparities exist with telemedicine use during the pandemic. This study evaluated health disparities associated with telemedicine uptake during the COVID‐19 pandemic among cancer patients in a tertiary care academic medical center.

**Methods:**

This retrospective cohort study evaluated telemedicine use among adult cancer patients who received outpatient medical oncology care within a tertiary care academic healthcare system between January and September 2020. We used multivariable mixed‐effects logistic regression models to determine how telemedicine use varied by patient race/ethnicity, primary language, insurance status, and income level. We assessed geospatial links between zip‐code level COVID‐19 infection rates and telemedicine use.

**Results:**

Among 29,421 patient encounters over the study period, 8,541 (29%) were delivered via telemedicine. Several groups of patients were less likely to use telemedicine, including Hispanic (adjusted odds ratio [aOR] 0.86, *p* = 0.03), Asian (aOR 0.79, *p* = 0.002), Spanish‐speaking (aOR 0.71, *p* = 0.0006), low‐income (aOR 0.67, *p* < 0.0001), and those with Medicaid (aOR 0.66, *p* < 0.0001). Lower rates of telemedicine use were found in zip codes with higher rates of COVID‐19 infection. Each 10% increase in COVID‐19 infection rates was associated with an 8.3% decrease in telemedicine use (*p* = 0.002).

**Conclusions:**

This study demonstrates racial/ethnic, language, and income‐level disparities with telemedicine use, which ultimately led patients with the highest risk of COVID‐19 infection to use telemedicine the least. Additional research to better understand actionable barriers will help improve telemedicine access among our underserved populations.

## Graphical abstract

At a tertiary care academic medical center, Hispanic, Asian, Spanish‐speaking, low‐income, and patients with Medicaid insurance were less likely to utilize telemedicine. Additionally, lower rates of telemedicine use were found in zip codes with higher rates of COVID‐19 infection. As such, there were significant racial/ethnic, language, and income‐level disparities with telemedicine use, which ultimately led cancer patients with the highest risk of COVID‐19 infection to use telemedicine the least.

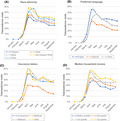

## 1 INTRODUCTION

In March of 2020, in response to the COVID‐19 pandemic, the Centers for Medicare & Medicaid Services (CMS) dramatically loosened regulations surrounding telemedicine use.^1^ These changes enabled healthcare systems across the US to rapidly implement telemedicine, or drastically scale up existing telemedicine workflows.^2^, ^3^ The use of telemedicine during the pandemic has clear advantages by reducing the risk of exposure to vulnerable patients and protecting the healthcare workforce. Within the field of oncology, patients with cancer are at an increased risk of COVID‐19 infection, and if infected with COVID‐19, have worse outcomes,^4^ which makes oncology well‐suited to telemedicine during the pandemic. The American Society of Clinical Oncology issued guidance early in the pandemic encouraging the use of telemedicine in settings such as patient education and counseling, assessment of treatment adherence, follow‐up, survivorship and palliative care.^5^

The COVID‐19 pandemic has exposed and exacerbated substantial health disparities across racially and ethnically diverse communities as well as among low‐income individuals.^6^, ^7^, ^8^, ^9^, ^10^ The use of telemedicine has the capacity to reduce exposure risks for our most vulnerable patients, though existing research pre‐COVID‐19 highlights the potential for inequity with access and use of telemedicine among those from disadvantaged sociodemographic backgrounds, limited English proficiency, and lower income households.^11^, ^12^ The rapid expansion of telemedicine across the country was essential in helping reduce exposure while retaining the ability to deliver cancer care. Some consider telemedicine a “silver lining” of the pandemic,^13^ yet we lack an understanding of whether disparities exist with telemedicine implementation during COVID‐19. An improved understanding of populations at risk of inequity with telemedicine will help raise awareness among providers and healthcare administrators to guide resource allocation, planning, and inform future interventions aimed at improving access. The purpose of this study was to evaluate health disparities in telemedicine implementation in a large tertiary care health network, with a focus on evaluating disparities by race, ethnicity, language, health insurance status and patient income. In addition, we assessed the geospatial link between regional telemedicine uptake and community COVID‐19 infection rates.

## 2 METHODS

### 2.1 Study environment

This study evaluated the patterns of outpatient oncology care and telemedicine use among all cancer patients receiving care within the University of California San Diego Health System. Within this network, oncology patients receive care through Moores Cancer Center, a National Cancer Institute (NCI) designated Comprehensive Cancer Center. Patients also receive care through three regional facilities including one urban hospital, and two separate suburban satellite facilities. All facilities were located in San Diego County, with a population of 3.3 million, representing an racially and ethnically diverse county with a large Hispanic population (32%).^14^ The county borders Mexico to the south and includes urban and suburban regions concentrated in the western portion of the county, and semi‐rural or rural designations in the east.^15^

### 2.2 Study cohort

This study identified all cancer patients over 18 years of age who received outpatient oncology care within UC San Diego Health between January 1 to September 30, 2020. This includes a period before and after telemedicine expansion at UC San Diego Health which occurred on March 16^th^, 2020 (see below for details). We included only patient encounters with medical oncology physicians and did not include encounters with surgery, radiation oncology or other cancer‐related providers. Encounters include initial consultations with medical oncologists, on‐treatment assessment, follow‐up post‐treatment encounters, and visits involving survivorship/palliative care. We did not include encounters where patients underwent procedures or received infusions.

### 2.3 Telemedicine implementation

UC San Diego Health had an established telemedicine infrastructure in place prior to COVID‐19,^16^ though this was limited to select services lines, such as tele‐stroke or tele‐psychiatry, and telemedicine was not utilized within oncology. With the onset of the COVID‐19 pandemic in March 2020, UC San Diego Health rapidly expanded telemedicine capacity across the network. A full description of the administrative and operational logistic details related to this telemedicine expansion was published previously.^17^ Briefly, individual disease teams were given discretion to identify patients potentially eligible for telemedicine. Telemedicine video interactions between patients and providers relied on a secure video application through Epic Systems (Epic Systems Corporation). This video application on the patient side required access to a smartphone or tablet with internet access and required patients to download, install, and register for the Epic MyChart application. The patient disease teams contacted eligible patients to determine if they had access to a smartphone or tablet, as well as internet access. Patient instructions were posted online, and written instructions were made available in English, Spanish, Chinese, and Arabic. The Epic MyChart online application has either an English or Spanish language interface. For limited English proficiency patients who needed an interpreter, a third‐party interpreter was included in the telemedicine visit. Patients unable to access video were scheduled for telephone visits with providers at the discretion of the individual disease teams. The telemedicine expansion within oncology launched on Monday, March 16th, 2020.

### 2.4 Study variables and outcomes

Patient demographics extracted from the electronic medical record system included patient gender, age, race/ethnicity, marital status, cancer type, cancer stage, preferred language, and insurance status. Median household income was estimated with a linkage to U.S. Census data at the zip‐code level. Cancer stage was not included in our primary due to a large fraction of patients missing stage data (66% with missing stage). However, we incorporated stage into a sensitivity analysis (on the cohort of patients with known stage), which did not influence our results (data not shown). The primary study outcome was the visit encounter type as noted within the electronic medical record, classified as an in‐person office visit, or a telemedicine encounter. Telemedicine encounters included either a video or telephone visit between the patient and provider.

### 2.5 Statistical analysis

To determine predictors of telemedicine use, we used a multivariable mixed effects logistic regression model. Given that individual patients completed more than one visit over the study period, this analytic approach allowed us to account for clustering at the patient level. Variables in the multivariable model were chosen a priori and included our four main variables of interest of race/ethnicity, preferred language, insurance status, and household income level. Multivariable models also included potential confounders including patient sex, age at visit, and cancer type.

In our geospatial analysis, we defined the regional telemedicine rate as the total number of telemedicine visits per zip code divided by the total number of encounters (in person and telemedicine) in that zip code. Regional COVID‐19 infection rates were obtained from the county of San Diego and expressed as the total number of COVID‐19 cases in that zip code per 100,000 individuals.^18^ More detailed address data was available for patients, though we did not have access to more granular geographic data regarding COVID‐19 cases, therefore we used zip code as the link with this analysis. To assess the association between regional telemedicine rates and regional COVID‐19 infection rates, we used a linear regression, weighted to account for differences in the number of total patient encounters per zip code. Statistical analyses were performed using SAS, version 9.4 (SAS Institute Inc, Cary, N.C.). All statistical testing was 2‐tailed, with *p * <  0.05 designated as statistically significant.

## 3 RESULTS

**FIGURE 1:**
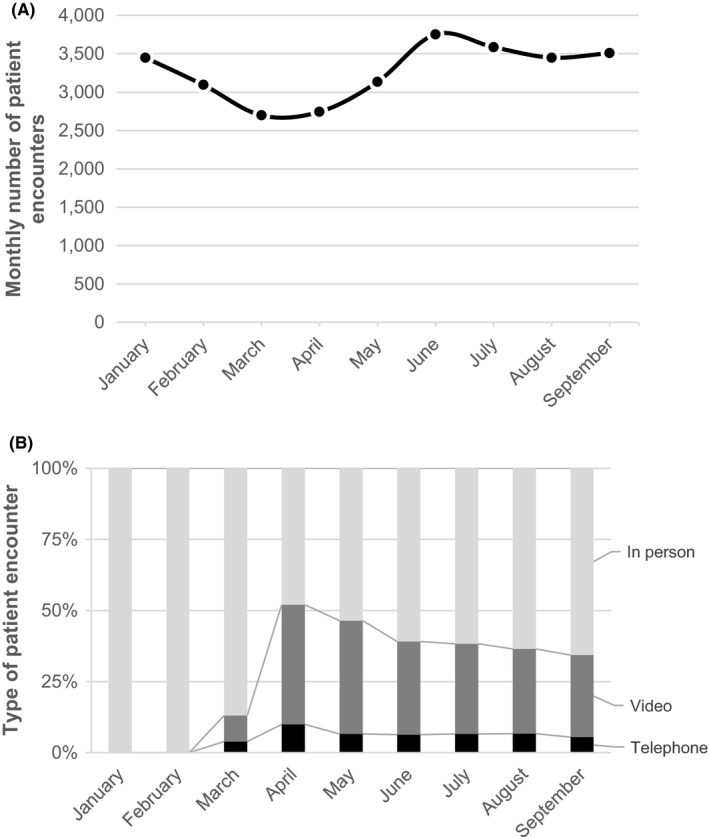
Total number of oncology visit and telemedicine trends. The top panel (1A) demonstrates the number of patient encounters between January and September 2020. The bottom panel (1B) shows the percentage of visits conducted in person (light gray bars), over video (dark gray bars), or telephone (black bars) over the same study period

Between January 1 and September 30, 2020, we identified 29,421 patient encounters among 8,997 patients treated by 43 providers, with characteristics of patient encounters included in Table 1. The total number of monthly patient encounters varied over the study period, decreasing in March and April, before rebounding to pre‐COVID numbers by June (Figure 1A). Despite the decrease in total patient encounters, there was no substantial change in the proportion of patient encounters by race/ethnicity, language, insurance status or income across the study period (Figure S1).

**TABLE 1:** Patient demographics

Characteristic	Number (%)
Sex	
Male	13,261 (45)
Female	16,160 (55)
Age at visit, years	
<55	7,600 (26)
55–64	7,630 (26)
65–74	8,280 (28)
≥75	5,911 (20)
Cancer site	
Gastrointestinal	6,551 (22)
Breast	7,881 (27)
Genitourinary	4,193 (14)
Lymphoma/leukemia	2,919 (9.9)
Lung	2,898 (9.9)
Head and neck	1,531 (5.2)
Gynecologic	428 (1.5)
Central nervous system	117 (0.4)
Other	2,903 (9.9)
Marital status	
Single	5833 (20)
Married	17479 (60)
Divorced	2660 (9.1)
Other	3145 (11)
Race and ethnicity	
Non‐Hispanic White	17,821 (61)
Hispanic	5,499 (19)
Non‐Hispanic Asian	3,007 (10)
Non‐Hispanic Black	1,201 (4.1)
Other	1,893 (6.4)
Preferred language	
English	25,561 (87)
Spanish	2,489 (8.5)
Other	1,371 (4.7)
Median household income	
Bottom quartile	2,306 (7.8)
2nd quartile	2,388 (8.1)
3rd quartile	9,912 (34)
Top quartile	14,815 (50)
Insurance	
Commercial	16,595 (56)
Medicaid	1,488 (5.1)
Medicare	10,868 (37)
Other	470 (1.6)

The use of telemedicine as a percentage of the total number of visits increased in March 2020, peaking by April 2020, when telemedicine accounted for 52% of all oncology patient visits (Figure 1B). After the peak in April, telemedicine use decreased and by September 2020, telemedicine use stabilized accounting for 34% of all encounters.

**FIGURE 2:**
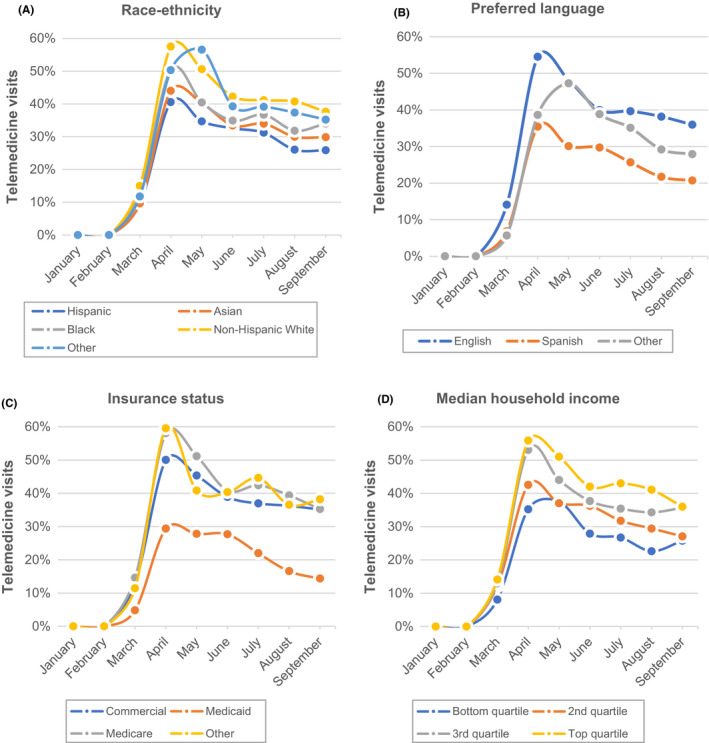
Trends in telemedicine use by patient characteristics. The plots in this figure demonstrate trends in telemedicine use between January and September 2020 stratified by patient race‐ethnicity (2A), preferred language (2B), patient insurance status (2C), and zip‐code level median household income (2D)

Over the study period, 8,541 encounters occurred via telemedicine, of which 7,061 (83%) occurred via video, and 1,480 (17%) over the telephone. The unadjusted monthly rates of telemedicine use over the study period varied by race‐ethnicity, language, insurance status, and median household income (Figure 2). Gaps in telemedicine use started early after telemedicine expansion in March/April and persisted through the end of the study in September.

**FIGURE 3:**
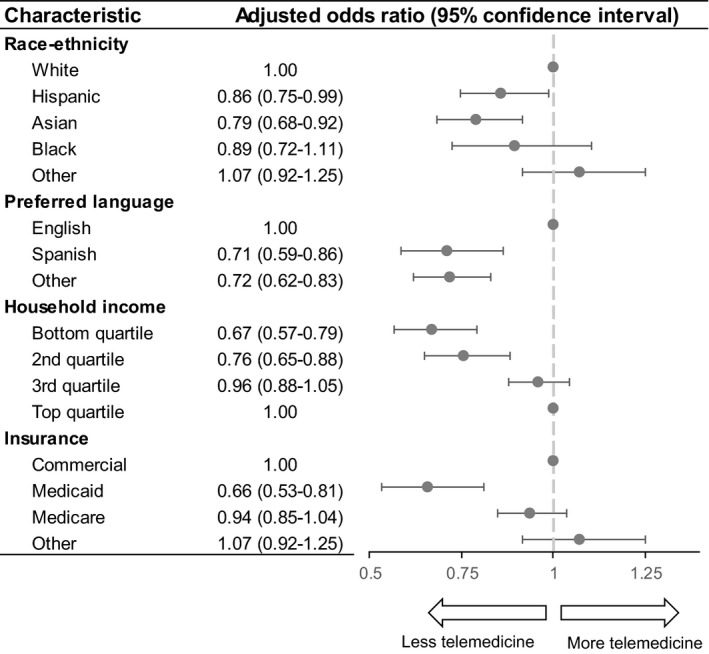
Multivariable analysis of telemedicine use. This figure represents the results of a multivariable mixed‐effects logistic regression to predict the use of telemedicine (defined as either video or telephone visits). The multivariable model included variables of race/ethnicity, preferred language, insurance status and household income level. Multivariable models also included potential confounders including patient sex, age at visit, and cancer type

Differences by race and ethnicity, language, insurance status, and income were shown in multivariable analysis controlling for other factors (Figure 3). Compared to non‐Hispanic white patients, we found that Hispanic patients had 14% lower odds of using telemedicine (*p* = 0.03), and non‐Hispanic Asian patients had 21% decreased odds of using telemedicine (*p* = 0.002). Compared to non‐English speakers, Spanish‐speaking patients had 29% decreased odds (*p* = 0.0006), and patients speaking languages other than English or Spanish had 28% decreased odds (*p* = 0.007) of using telemedicine. Compared to the top (4th) income quartile, those in the 2nd quartile had 24% decreased odds (*p* = 0.0003), and those in the bottom (1st) quartile had 33% decreased odds (*p *< 0.0001) of using telemedicine. Compared to patients with private insurance, those with Medicaid had 34% decreased odds (*p *< 0.0001) of using telemedicine. Table S1 demonstrates complete results from the multivariable regression.

**FIGURE 4:**
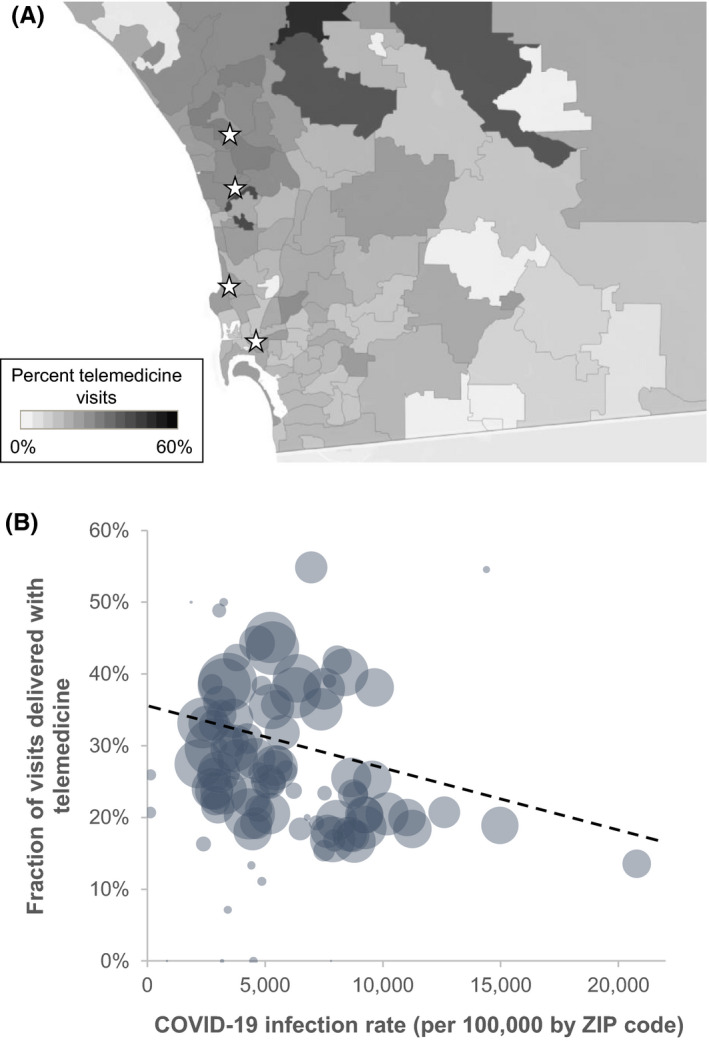
Geospatial distribution of telemedicine visits and association with COVID‐19 infection rates. The top plot (3A) demonstrates the zip‐code level telemedicine rate in San Diego County by individual zip code. Telemedicine rate was defined as the number of telemedicine encounters divided by the total number of in person or telemedicine encounters. The white stars represent outpatient oncology clinics. The bottom plot (3B) demonstrates the relationship between the zip‐code level telemedicine rate and the zip code rates of COVID‐19. Each bubble represents an individual zip code and the area of the bubble correlates with the total number of patient encounters in that zip code. The black dashed line represents the trend between COVID‐19 infection rates and telemedicine rates

Geospatial comparisons of telemedicine use demonstrated heterogeneity across San Diego county (Figure 4A) with unadjusted zip‐code level rates of telemedicine use ranging from 0% to 55%. Across San Diego county, there were 213,168 cases of COVID‐19, and the overall COVID‐19 infection rate across the county was 6,882 per 100,000, or 6.88%. Lower rates of telemedicine use were found in zip codes with higher COVID‐19 infection rates (*p* = 0.002; Figure 4B). Each 10% increase in COVID‐10 infection rates by zip code was associated with an 8.3% decrease in telemedicine use.

## 4 DISCUSSION

The COVID‐19 pandemic has disproportionately impacted underserved populations, with substantially higher coronavirus infection rates and increased COVID‐19 hospitalizations and mortality rates among Black and Hispanic/Latino populations.^7^, ^10^, ^19^, ^20^, ^21^ The current study found substantial inequity in the utilization of telemedicine during the COVID‐19 pandemic at a tertiary care academic medical center. We found lower rates of telemedicine use among our minority and underserved populations that cut across race, ethnicity, language, income level, and insurance. Furthermore, the rates of telemedicine use were lowest in zip codes with the highest COVID‐19 infection rates. The patients at highest risk of contracting COVID‐19 potentially have the most to gain from telemedicine during the pandemic, yet we found that telemedicine use was lowest in these high‐risk groups.

Telemedicine inherently relies on technology, and when considering inequities in telemedicine use one must consider the divide in *digital inclusion*, which involves both patient access to technology as well as the digital literacy of patients.^22^ Research demonstrates that technology ownership and digital literacy vary by race, ethnicity, preferred language, and income.^23^, ^24^, ^25^, ^26^, ^27^ At our institution, telemedicine was preferentially delivered via video, which required a smartphone or tablet with internet access. Existing research demonstrates similar smartphone ownership between white and Hispanic patients, yet lower rates of home broadband access for Hispanic populations.^28^ Similarly, research demonstrates a clear digital divide among lower income individuals and those with limited English proficiency, even with the availability of third‐party interpreter services.^24^, ^29^, ^30^ We found lower rates of telemedicine use among individuals residing in low‐income zip codes, those covered by Medicaid, and among our non‐English speaking patients. This study demonstrates a link between disparities in social determinants of health (income, language, and literacy skills) and utilization of telemedicine. The tightening connection between technology and healthcare has led to calls for digital access and availability to be considered a social determinant of health.^31^

When considering the patterns of telemedicine use in our study access barriers do not explain all our observed disparities. For example, Asians have higher rates or smartphone ownership and home broadband access,^25^ yet we found this cohort of patients to have lower rates of telemedicine. However, Asian‐Americans face other barriers to healthcare delivery and the decreased use of telemedicine could stem from differences in health literacy, communication, or cultural preferences toward healthcare.^32^, ^33^, ^34^ This brings about the important concept of patient perceptions toward telemedicine, and how this may impact telemedicine disparities. This current study did not evaluate patient preferences toward telemedicine, though research on telemedicine acceptance during COVID‐19 among cancer patients suggests nearly half of patients who decline telemedicine do so because of a preference for face‐to‐face visits.^35^ Additionally, research prior to COVID‐19 demonstrates that perceptions of telemedicine vary by race and ethnicity. Focus group interviews with Hispanic individuals point to potential concerns about adequacy of telemedicine to provide effective healthcare, and also concerns about privacy, confidentiality, and security.^36^ Trust in telemedicine is another consideration. A fraction of patients either would not trust a diagnosis made via telemedicine or would trust it less than one made by a provider in person.^37^ The question of trust deserves additional scrutiny given that we lack an understanding of whether trust in telemedicine varies by a patient's race, ethnicity, language, or socioeconomic status.

This study evaluated inequity in telemedicine among oncology patients during the COVID‐19 pandemic. Understanding these disparities during the pandemic will help us more equitably utilize telemedicine as we emerge from the pandemic. To what extent we use telemedicine post‐pandemic will inherently depend on several factors, including federal and state health policy changes, and the stability of reimbursement rates. Despite this future uncertainty, many factors suggest that telemedicine use will continue beyond the pandemic. Importantly, CMS has recently signaled continued support for telemedicine that will extend past the pandemic.^38^ A substantial body of research prior to COVID‐19 demonstrates efficacy and safety with telemedicine across a range of specialties,^39^, ^40^ and research during COVID‐19 demonstrates the ability of telemedicine to deliver high quality oncology care.^41^ Studies among cancer patients demonstrate high patient satisfaction rates with telemedicine, in large part due to the reduced travel burden and decreased costs.^42^, ^43^ It is important to consider that travel burden and cost disproportionately impact our racial/ethnic minority communities as well as those with lower income. This emphasizes the fact that culturally and linguistically tailored telemedicine could help reduce health disparities for our vulnerable cancer patients. As we emerge from the pandemic, the role of telemedicine in oncology will become more firmly established. This study highlights the future need to deliberately consider equitable implementation of telemedicine to avoid increasing health disparities.

There are limitations with this study worth noting. This study involved a single tertiary care cancer center in mostly urban areas containing a large Spanish‐speaking population, and we cannot assess whether these findings generalize to other healthcare environments or different population demographics. Recent research in healthcare environments outside of oncology have observed disparities in telemedicine during COVID‐19,^11^ though future population‐based research is needed to more thoroughly assess for the presence of widespread inequity. Another limitation of this study relates to the retrospective observational design. We could not assess whether patients were offered telemedicine and refused. We also could not assess the influence of system‐level factors such as whether provider teams introduced bias through preferentially offering telemedicine to certain groups of patients. We found that the use of telemedicine varied by cancer subtype, however, the limited number of patients with specific cancer prevented us from a well‐powered analysis evaluating whether our observed disparities held across cancer types. This study did not incorporate details specific to a patient's medical condition, therefore we cannot assess appropriateness of telemedicine use. Along these lines, underlying health disparities in cancer presentation, treatment, outcomes, as well as disparities in comorbidity, could all influence a provider team's decision of whether telemedicine is suitable for a given patient. Health disparities in telemedicine could in part reflect the well‐documented racial, ethnic, language, and socioeconomic health disparities associated with cancer.^44^ Additional research—both quantitative and qualitative—is needed to better understand the full picture of health disparities in telemedicine.

COVID‐19 has rapidly transformed how patients access their healthcare, with a dramatic increase in the use of telemedicine. This study demonstrates disparities in telemedicine use among racial and ethnic minorities, along with inequities that extend across social determinants of health including language, income level, and health insurance status. Ultimately these disparities translate into patients with the highest risk of COVID‐19 infection using telemedicine the least. Results of this study emphasize the need for individual healthcare systems to look at health equity with their own experience with telemedicine implementation. Additional research into disparities in telemedicine will help identify actionable barriers required to improve access to our underserved populations.

## CONFLICT OF INTEREST

JM reports receiving compensation for consulting from Boston Consulting Group. Dr. Patel receives scientific advisory income from: Amgen, AstraZeneca, Beigene, Bristol‐Myers Squibb, Certis, Eli Lilly, Genentech, Illumina, Merck, Pfizer, Rakuten, Tempus. Dr. Patel's university receives research funding from: Bristol‐Myers Squibb, Eli Lilly, Incyte, AstraZeneca/MedImmune, Merck, Pfizer, Roche/Genentech, Xcovery, Fate Therapeutics, Genocea, Iovance.

## AUTHOR CONTRIBUTIONS

The authors confirm contribution to the manuscript as follows: Study conceptualization and design: AQ, MS, JM. Data curation and formal analysis: AQ, VN, LA, FP, BN, JM. Visualization: AQ, VN, JM. Writing—original draft: AQ, JM. Writing—review and editing: AQ, MS, PV, SP, MM, JM. All authors reviewed the results and approved the final version of the manuscript.

## ETHICAL APPROVAL STATEMENT

The authors are accountable for all aspects of the work in ensuring that questions related to the accuracy or integrity of any part of the work are appropriately investigated and resolved. All procedures performed in studies involving human participants were in accordance with the ethical standards of the institutional and/or national research committee(s) and with the Helsinki Declaration (as revised in 2013). Written informed consent was obtained from the patient.

## Supporting information

Fig S1

Table S1

## Data Availability

Cancer Medicine expects that data supporting the results in the paper will be archived in an appropriate public repository. Authors are required to provide a data availability statement to describe the availability or the absence of shared data. When data have been shared, authors are required to include in their data availability statement a link to the repository they have used, and to cite the data they have shared. Whenever possible the scripts and other artifacts used to generate the analyses presented in the paper should also be publicly archived. If sharing data compromises ethical standards or legal requirements then authors are not expected to share it.
