# Supplementary material for: Disparities in telemedicine during COVID‐19
Source: Cancer Med. 2022 Jan 5;11(4):1192–201. doi: 10.1002/cam4.4518 (PMC8855911; doi:10.1002/cam4.4518)
Supplement: Supplementary file 1 — Fig S1 [file CAM4-11--s002.docx]

**Supplemental Material**

**Supplemental Figure 1. Trends in visit demographics.** This figure demonstrates the trends in patient demographics for oncology visits between January and September 2020. This includes visit trends by patient race/ethnicity (1A), preferred language (1B), insurance status (1C), and median household income in quartiles (1D).
