# Supplementary material for: Disparities in telemedicine during COVID‐19
Source: Cancer Med. 2022 Jan 5;11(4):1192–201. doi: 10.1002/cam4.4518 (PMC8855911; doi:10.1002/cam4.4518)
Supplement: Supplementary file 2 — Table S1 [file CAM4-11--s001.docx]

**Supplemental Table 1.** This table represents the full results of a multivariable mixed-effects logistic regression to predict the use of telemedicine (defined as either video or telephone visits).

| **Characteristic** | | **Adjusted odds ratio for telemedicine use (95% CI)** | **p-value** |
| --- | --- | --- | --- |
| Sex | |  |  |
|  | Male |  |  |
|  | Female | 1.27 (1.15-1.41) | <0.0001 |
| Age at visit | |  |  |
|  | <55 | 1 |  |
|  | 55-64 | 1.01 (0.90-1.13) | 0.83 |
|  | 65-74 | 1.23 (1.09-1.40) | 0.001 |
|  | ≥75 | 1.35 (1.17-1.55) | <0.0001 |
| Race and ethnicity | |  |  |
|  | Non-Hispanic White | 1 |  |
|  | Hispanic | 0.87 (0.76-1.00) | 0.044 |
|  | Non-Hispanic Asian | 0.78 (0.68-0.91) | 0.001 |
|  | Non-Hispanic Black | 0.93 (0.75-1.15) | 0.48 |
|  | Other | 1.08 (0.93-1.27) | 0.31 |
| Marital Status | | | |
|  | Married | 1 |  |
|  | Single | 0.85 (0.76-0.95) | 0.0002 |
|  | Divorced | 0.91 (0.79-1.04) | 0.15 |
|  | Other | 0.87 (0.76-1.04) | 0.06 |
| Cancer site | |  |  |
|  | Gastrointestinal | 1 |  |
|  | Breast | 0.35 (0.31-0.40) | <0.0001 |
|  | Genitourinary | 0.43 (0.38-0.50) | <0.0001 |
|  | Lymphoma/leukemia | 1.21 (1.06-1.39) | 0.006 |
|  | Lung | 0.43 (0.37-0.51) | <0.0001 |
|  | Head and neck | 0.44 (0.35-0.53) | <0.0001 |
|  | Gynecologic | 0.60 (0.43-0.84) | 0.003 |
|  | Central nervous system | 0.73 (0.42-1.25) | 0.24 |
|  | Other | 0.72 (0.62-0.83) | <0.0001 |
| Preferred language | |  |  |
|  | English | 1 |  |
|  | Spanish | 0.70 (0.58-0.85) | 0.0004 |
|  | Other | 0.75 (0.61-0.93) | 0.008 |
| Median household income | |  |  |
|  | Bottom quartile | 0.69 (0.58-0.81) | <0.0001 |
|  | 2nd quartile | 0.78 (0.67-0.91) | 0.002 |
|  | 3rd quartile | 0.98 (0.90-1.07) | 0.59 |
|  | Top quartile | 1 |  |
| Insurance | |  |  |
|  | Commercial | 1 |  |
|  | Medicaid | 0.67 (0.54-0.83) | 0.0002 |
|  | Medicare | 0.94 (0.85-1.04) | 0.24 |
|  | Other | 1.05 (0.79-1.41) | 0.74 |
